# Draft genome sequence of *Burkholderia vietnamiensis* FBCC-B8049 from freshwater environments with antifungal and plant growth-promoting activities

**DOI:** 10.1128/mra.01272-25

**Published:** 2026-02-03

**Authors:** Gil Han, Chang Soo Lee

**Affiliations:** 1 Fungi Research Division, Nakdonggang National Institute of Biological Resources 443035, Sangju-si, Gyeongsangbuk-do, South Korea; University of Manitoba, Winnipeg, Manitoba, Canada

**Keywords:** draft genome, *Burkholderia vietnamiensis*, freshwater environment, antifungal agents, plant growth promotion

## Abstract

We report on the draft genome sequence of *Burkholderia vietnamiensis* FBCC-B8049 isolated from freshwater environments in Korea. The 7.26-Mb genome encodes 6,567 predicted genes and provides insights into its antifungal and plant growth-promoting activities.

## ANNOUNCEMENT

*Burkholderia vietnamiensis* strain FBCC-B8049 was isolated from freshwater collected near a water reservoir in Sangju-si, Gyeongsangbuk-do, South Korea (36.4173 N, 128.2696 E) on 4 April 2021. The strain exhibits strong antagonistic activity against plant pathogenic fungi, including *Colletotrichum acutatum* and *Fusarium oxysporum*, and demonstrates plant growth-promoting activities (1).

The strain was initially isolated from freshwater samples by serial dilution on Reasoner’s 2A (R2A) agar, but cultivation for genomic DNA extraction was performed in tryptic soy broth (TSB). Based on our previous study (1), cells were harvested at exponential phase for genomic DNA extraction using the Wizard Genomic DNA Purification Kit (Promega, Madison, WI, USA). The same extract was used for sequencing on both the Illumina and PacBio platforms (Macrogen, Inc., South Korea).

For long-read sequencing, high-molecular-weight DNA was used for PacBio Revio library preparation with the SMRTbell Prep Kit 3.0 (PacBio) following the manufacturer’s protocol. Sequencing of HiFi read statistics on the PacBio Revio platform yielded 64,260 reads totaling 520.2 Mbp, with an N50 of 9,149 bp, an average read length of 8,094 bp, and an average quality of Q39. No intentional mechanical shearing and size-selection step was performed prior to sequencing.

For short-read sequencing, paired-end libraries (2 × 150 bp) were prepared using the Illumina TruSeq DNA PCR-Free Kit (Illumina, San Diego, CA) according to the manufacturer’s instructions. Sequencing on the Illumina NovaSeq 6000 platform generated 4.6 Gbp of raw reads, which were quality-filtered with Trimmomatic v0.38 (2), corresponding to an approximate sequencing depth of ~634×. Hybrid *de novo* assembly was performed with Raven v1.8.1, and the assembly was polished with Pilon v1.22 (3, 4).

The hybrid assembly generated five contigs totaling 7,264,122 bp, with an N50 of 2,177,826 bp and a guanine and cytosine (GC) content of 66.7%. These included four circular contigs (3,409,684, 2,177,826, 388,327, and 20,204 bp) and one linear contig (1,268,081 bp). For one contig, the presence of large inverted repeats at the contig ends precluded confident circularization, and no overlap trimming or rotation was performed. BUSCO v5.1.3 (bacteria_odb10) was used to assess assembly completeness based on universal single-copy orthologs, indicating 99.19% completeness (5). Annotation was performed using Prokka v1.14.6, a rapid prokaryotic genome annotation pipeline (6) (Table 1). ANI analysis with pyani v0.2.7 confirmed that FBCC-B8049 belongs to *B. vietnamiensis*, showing 99.16% ANI and 88.16% coverage with the reference strain *B. vietnamiensis* Arc4 (7).

**TABLE 1:** Genome assembly statistics of *Burkholderia vietnamiensis* FBCC-B8049

	*B. vietnamiensis* FBCC-B8049
No. of contigs	5 (four circular, one linear)
Total size (bp)	7,264,122
GC content (%)	66.7
Sequencing depth	71.61
Largest contig (bp)	3,409,684
N50 (bp)	2,177,826
Gene	6,567
Coding sequences (CDSs)	6,471
rRNAs	18
tRNAs	77
tmRNA	1

The FBCC-B8049 genome harbors key gene clusters associated with its beneficial functions, as inferred from genome annotation. These include the *nif* gene cluster for nitrogen fixation and its activator gene *nifA* (8). The *pqq* operon responsible for pyrroloquinoline quinone (PQQ) and gluconic acid biosynthesis contributes to phosphate solubilization together with *pstS*, *pstC*, *pstA*, and *pstB* (9). Genes involved in indole-3-acetic acid (IAA) biosynthesis, including tryptophan 2-monooxygenase, tryptophan decarboxylase, and nitrilase, were present (9). Several genes related to siderophore uptake, such as ABC type transporter, were also found (9). Taken together, these gene clusters highlight the multi-functional genetic mechanisms underlying the antagonistic and plant growth-promoting potential of FBCC-B8049 (1).

## Data Availability

The BioProject accession number is PRJNA1332138, and the BioSample accession number is SAMN51723525. Raw sequencing reads have been deposited in the Sequence Read Archive (SRA) under accession numbers SRR35532367 and SRR35539707.
